# Predictors of Active Extravasation and Complications after Conventional Angiography for Acute Intraabdominal Bleeding

**DOI:** 10.3390/jcm6040047

**Published:** 2017-04-18

**Authors:** Zachary M. Haber, Hearns W. Charles, Joseph P. Erinjeri, Amy R. Deipolyi

**Affiliations:** 1 School of Medicine, New York University, 550 1st Avenue, New York, NY 10016, USA; zachary.haber1@gmail.com; 2 South Florida Vascular Associates, Coconut Creek, FL 33073, USA; tblouverture@gmail.com; 3 Interventional Radiology Service, Memorial Sloan Kettering Cancer Center, New York, NY 10065, USA; erinjerj@mskcc.org

**Keywords:** hemorrhage, angiography, contrast-induced nephropathy, hematocrit, GFR

## Abstract

Conventional angiography is used to evaluate and treat possible sources of intraabdominal bleeding, though it may cause complications such as contrast-induced nephropathy (CIN). The study’s purpose was to identify factors predicting active extravasation and complications during angiography for acute intraabdominal bleeding. All conventional angiograms for acute bleeding (January 2013–June 2015) were reviewed retrospectively, including 75 angiograms for intraabdominal bleeding in 70 patients. Demographics, comorbidities, vital signs, complications within one month, and change in hematocrit (ΔHct) and fluids and blood products administered over the 24 h prior to angiography were recorded. Of 75 exams, 20 (27%) demonstrated extravasation. ΔHct was the only independent predictor of extravasation (*p =* 0.017), with larger ΔHct (−17%) in patients with versus those without extravasation (–1%) (*p =* 0.01). CIN was the most common complication, occurring in 10 of 66 angiograms (15%). Glomerular filtration rate (GFR) was the only independent predictor (*p =* 0.03); 67% of patients with GFR < 30, 29% of patients with GFR 30–60, and 8% of patients with GFR > 60 developed CIN. For patients with intraabdominal bleeding, greater ΔHct decrease over 24 h before angiography predicts active extravasation. Pre-existing renal impairment predicts CIN. Patients with large hematocrit declines should be triaged for rapid angiography, though benefits can be weighed with the risk of renal impairment.

## 1. Introduction

Acute intraabdominal hemorrhage carries significant morbidity and mortality. In patients with gastrointestinal bleeding (GIB), mortality rates range from 8% to 14% and increase to 20–40% in episodes of massive bleeding [1,2,3,4,5]. Depending on the source of bleeding, several diagnostic and therapeutic procedures exist for evaluation, such as endoscopy, conventional angiography, tagged red blood cell (RBC) scans, and arterial phase multi-detector row helical computed tomography (CTA) [6,7,8]. Conventional angiography is used for many etiologies of intraabdominal bleeding due to its ability to both locate and treat active hemorrhage [7,9,10,11,12]. Super-selective embolization performed during the angiographic examination is an effective treatment for intraabdominal hemorrhage with high success (80–100%) and low recurrence rates (14–29%) [13,14]. However, there are multiple limitations with conventional angiography, including suboptimal detection rates and potential complications. Studies on mesenteric angiography in GIB report clinical sensitivity rates ranging between 22% and 87% [7,15]. Experimentally, it has been shown that for contrast extravasation to be detectable, bleeding must be at a rate of at least 0.5 cc/min [16]. It is unclear how to apply this number clinically, since the actual bleeding rate is unknown. Rather, clinical management is determined by observable features such as vital signs and laboratory values.

Conventional angiography has associated risks. For example, contrast-induced nephropathy (CIN) occurs in fewer than 1% of all patients receiving intravenous contrast, but in 12–27% of patients with pre-existing renal dysfunction [17]. More than 5% of trauma patients undergoing contrast-enhanced CT develop CIN, suggesting that bleeding and/or hypotension increase risk. CIN is associated with increased morbidity, mortality, and healthcare costs [18] and should be avoided whenever possible. The challenge for the interventional radiologist consulted in the evaluation of acute bleeding is to balance the potential for detecting a treatable vascular focus with the possibility of complications including CIN. The goal of this study was to determine clinical predictors to assist in triaging patients.

## 2. Experimental Section

This retrospective study was performed at a single academic tertiary care center. Patients were identified by review of interventional radiology records of procedures performed for acute bleeding between January 2013 and June 2015. This yielded 102 arteriograms that were performed during the study period for an indication of acute intraabdominal hemorrhage. Six were excluded as there was no recorded data before and after the procedure. The remaining 96 procedures were performed in 86 patients; one patient had three arteriograms and eight patients had two. For the purpose of analysis, each arteriogram was considered as a separate independent event.

Among the 96 arteriograms, 21 were excluded from the analysis of variables contributing to active extravasation, as they were not for intraabdominal bleeding (retroperitoneal or urinary tract: 14; hematoma of an extremity: 3; hemoptysis: 4). Therefore, 75 angiograms were studied, with indications including intra-hepatic bleeding (4), splenic bleeding (6), upper GIB (21), lower GIB (20), GIB of uncertain location (5), uterine/vaginal bleeding (12), pancreatic bleeding (2), and intraabdominal bleeding from other causes (5). These 75 arteriograms were performed in 70 patients (one patient had three procedures and two patients had two). Among these 70 patients, 44 were men and 26, women. The average age of patients was 59 years (range 21–92 years). 

CIN was defined as a 25% increase in creatinine from baseline or a 0.5 mg/dL increase in absolute creatinine 48–72 h from contrast administration. CIN was assessed for all studies, including the additional 21 studies performed for other sources of hemorrhage such as hemoptysis and urinary tract bleeding. Of the 96 arteriograms performed for hemorrhage, 66 had data regarding renal function before and after the procedure and were included in the assessment for CIN.

Three types of anesthesia were used during angiographic procedures: local anesthesia and/or mild to moderate sedation provided by radiology nurses, monitored anesthesia care, or general anesthesia. The method of anesthesia was based on patient presentation and preference of both the attending interventional radiologist and anesthesiologist. Six board-certified, fellowship-trained interventional radiologists performed the procedures with or without the assistance of a fellow, resident, or medical student. 

Patients were positioned supine. Angiography was then performed using digital subtraction imaging and either Ultravist (Bayer, Whippany, NJ, USA) or Visipaque (GE Healthcare, Wauwatosa, WI, USA), most commonly via the right common femoral arterial approach, using a 5-Fr. introducer sheath (Cordis, Fremont, CA, USA). Target vessels were catheterized with a 5-Fr. catheter. If a bleeding site was identified, a 3-Fr. coaxial microcatheter was used to perform super-selective embolization with microcoils, Gelfoam pledgets (Pfizer, New York, NY, USA) or Avitene (Bard Davol Inc., Warwick, RI, USA) slurry, or 250–1000-micron microspheres, according to the preference of the interventionalist. Completion angiography was performed after embolization to confirm resolution of hemorrhage.

The primary indication for angiography, clinical variables, and follow-up were obtained by review of patients’ electronic medical records (Epic, Verona, WI, USA), including operative and clinic notes, radiology reports, and discharge summaries. Variables assessed included age, gender, history of congestive heart failure (CHF) or diabetes mellitus (DM), contrast amount and type. Urine output, number of units of packed red blood cells (pRBC), fluids, and hematocrit values were all assessed over the 24-h period prior to angiography. A percent change in hematocrit (ΔHct) was calculated between the initial and final hematocrit values from the 24-h period prior to angiography. The greatest rate of hematocrit drop on the patient’s current admission was determined through analysis of the last 15 Hct values prior to undergoing angiography. The mean arterial pressure (MAP), heart rate, whether vasopressors were administered, and last glomerular filtration rate (GFR) immediately preceding the study were also recorded.

A positive angiogram was defined as the presence of active contrast medium extravasation or a visualized blush. Two separate analyses were carried out. In the first analysis, groups were compared with regard to outcome of the angiographic study (positive versus negative for active extravasation). The second analysis compared studies resulting in CIN to those that did not. Statistical tests to compare the groups included *t*-tests (data with normal distribution) and Mann–Whitney U-tests (data without normal distribution) for continuous variables, chi-squared tests for categorical variables, and multiple logistic regression analysis, with *p* < 0.05 considered statistically significant. Statistical analysis was performed using GraphPad Prism 6 (GraphPad Software, Inc., La Jolla, CA, USA). 

This Institutional Review Board-approved and Health Insurance Portability and Accountability Act-compliant study was limited to the retrospective use of electronic medical records. 

## 3. Results

### 3.1. Predictors of Active Contrast Extravasation

Of the 75 angiograms included in the analysis of factors predicting active extravasation, 20 (27%) demonstrated active extravasation and 50 (73%) did not. Univariate analysis is presented in Table 1, and demonstrates that the only variables significantly different between groups were the number of units of packed red blood cells (pRBC) transfused and the percent change in hematocrit (ΔHct) over the prior 24 h. Patients with active extravasation on average had a –17% ΔHct, compared with −1% in patients with negative angiograms (*p =* 0.004). Patients with active extravasation received fewer pRBC (0.5 units) in the 24 h prior to arteriogram compared to patients without extravasation (1.6 units) (*p =* 0.02). 

Logistic regression was performed to determine variables predicting active extravasation, and included pRBC, total volume of fluids not including pRBC, mean arterial pressure (MAP) and heart rate (HR) just prior to angiography, treatment with vasopressors, and the ΔHct. The model was significant (χ^2^ = 12.8; *p =* 0.045); the only independent predictor of extravasation was ΔHct (*p =* 0.038); pRBC did not independently predict outcome (*p =* 0.08). Of 70 patients who had <20% ΔHct, 17 (24%) had active extravasation. Of 20 patients with ≥20% ΔHct, 10 (50%) had active extravasation (χ^2^ = 4.9; *p =* 0.027).

### 3.2. Predictors of Extravasation among Patients with GIB

Next, subgroup analysis was performed for all studies done for suspected GIB, which included 46 total examinations. Findings were similar when comparing patients with and without active extravasation. Multiple logistic regression including pRBC, total volume of fluids not including pRBC, mean arterial pressure (MAP) and heart rate (HR) just prior to angiography, treatment with pressors, and the ΔHct was statistically significant (χ^2^ = 15.4; *p =* 0.017). Again, ΔHct was the only independent predictor (*p =* 0.049), with a mean −21% ΔHct in the active extravasation group and −1% ΔHct in the patients without extravasation (*p =* 0.02). Table 2 describes the proportion of studies that showed active extravasation for different ΔHct. Though again, pRBC differed between groups on univariate analysis (U = 94; *p =* 0.006) with a larger amount of pRBC administered to patients without (2.2 units) than with (0.5 units) contrast extravasation; the pRBC administered to the patients in the 24 h before arteriogram was not an independent predictor in the regression (*p =* 0.10).

To understand why patients with active extravasation received fewer units of pRBC than patients without extravasation, the time from the greatest ΔHct to angiography was calculated as described previously for patients with GIB. Patients without active extravasation were assessed angiographically on average 34 h after the greatest ΔHct, compared to patients with extravasation who were assessed 21 h after the greatest ΔHct, though this difference was not statistically significant (*p =* 0.30). Power analysis demonstrates that 113 cases would be required to detect such a difference, with 80% power.

Prior to angiographic studies performed for GIB, 42 cases were preceded by endoscopy, 31 of which led to a negative angiogram, and the remaining 11 led to a study with extravasation. Of the 31 negative angiography cases, 19% had reported active bleeding on endoscopy; 58% suggested an etiology without active bleeding. Comparatively, of the 11 positive angiograms, 36% had active bleeding and 45% had a suggested lesion without bleeding on endoscopy. These differences were not significant (χ^2^ = 1.3; *p =* 0.52).

Prior to studies performed for GIB, 16 were preceded by CTA, 12 of which led to a negative angiogram, and four with extravasation. Of the four positive angiography studies, one (25%) had active extravasation suggested by CTA; one (25%) had a finding suggestive of an etiology but without bleeding, and two (50%) were negative despite subsequently showing extravasation on angiography. Negative arteriograms were preceded by three (25%) CTAs demonstrating active extravasation, seven (58%) with findings suggestive of an etiology but without bleeding, and two (17%) negative CTAs. These proportions were not significantly different (χ^2^ = 2.0; *p =* 0.37).

### 3.3. Adverse Events Following Angiography for Patients with Intraabdominal Bleeding

Adverse events, in addition to CIN, were assessed for all 75 studies for intraabdominal hemorrhage. There were two (2.6%) groin hematomas, and one (1.3%) arterial dissection precluding embolization despite the finding of active extravasation. The latter case was a source of lower GIB. Among the remaining 11 cases of GIB with active extravasation that were embolized successfully, two (18%) resulted in bowel ischemia noted on follow-up CT imaging that resolved without intervention.

Of the 96 arteriograms performed for hemorrhage, 66 had data regarding renal function before and after the procedure and were assessed regarding CIN. Of these, 10 (15%) led to CIN, and 56 (85%) did not. Table 3 compares these groups, and shows that age, gender, history of CHF or DM, contrast amount or type, pulse or MAP, and presence of active extravasation were not significantly different between patients who did and did not develop CIN. Variables with *p* < 0.10 (ΔHct and GFR) were included in a logistic regression analysis, with CIN as the outcome variable. This model was statistically significant (*p =* 0.008), though GFR was the only independently predictive variable (*p =* 0.03). Of patients with severe pre-existing renal impairment (GFR < 30), 67% developed CIN after arteriography; of patients with mild-moderate impairment (GFR 30–60), 29% developed CIN; of patients with normal renal function (GFR > 60), 8% developed CIN (Table 4).

## 4. Discussion

Since its development in the 1970s, conventional angiography with embolization has emerged as a critical tool in the diagnosis and treatment of acute intraabdominal bleeding [19,20,21]. When consulted to perform conventional angiography, the interventional radiologist must weigh the potential for finding a treatable vascular focus with the possibility of complications including contrast-induced renal impairment. The findings reported here demonstrate that the change in hematocrit over the 24 h preceding angiography was the only independent predictor of active extravasation, whereas vital signs, volume resuscitation, and transfusion volumes were not. On the other hand, pre-existing renal impairment was the only significant predictor of CIN in the context of angiography for acute bleeding.

The wide range of reported clinical sensitivities with conventional angiography has hindered the procedure’s potential clinical utility. Multiple studies have demonstrated an association with hemodynamic instability and number of units of pRBC transfused, whereas others have shown no such associations [13,22,23,24]. In this study, ΔHct over the previous 24 h was the only significant independent predictor of a positive angiographic study in all indications, a relationship that also held true in the specific context of GIB. For all patients including those with GIB, 20% decrease in hematocrit over 24 h appears to be a reasonable cutoff; patients with declines in excess of 20% are significantly more likely to have active contrast extravasation on conventional angiography, and therefore a visualized treatable vascular target. 

The findings presented here are similar to a prior report that a drop in hemoglobin exceeding 5 g/dL from previous admission correlated with a positive study (*p =* 0.02) [22]. However, many patients with intraabdominal hemorrhage have not had a prior admission, at least in the same institution, and therefore do not have prior laboratory assessment, limiting the applicability of that report. In contrast, the findings presented here relate to a single admission; most patients with GIB and other sources of intraabdominal hemorrhage undergo serial laboratory analysis. Thus, finding a significant change in hematocrit over the course of one day can be helpful to the interventional radiologist triaging a patient for angiography.

Other prior studies may have dismissed changes in hematocrit as powerful predictors for a positive angiographic study, due to this data not being readily available [24]. The data collected here were gathered from an electronic medical record; all medications, fluids, and blood products administered could not be given without an electronic order; similarly, vital signs and laboratory values were recorded with specific and reliable time stamps. This thorough electronic record therefore allows for the assessment of all variables’ potential contributions to predicting which patients would have contrast extravasation to indicate active bleeding. Prior studies suggested that the number of units of pRBC infused prior to angiography was the greatest predictor for a positive angiographic study [22,23,24]. In the current study, pRBC did not predict a positive study and there was an opposite trend with fewer units of pRBC associated with a positive angiographic study. This paradoxical finding could be due to differences in timing of the angiogram relative to active bleeding; longer delays between the bleeding and arteriogram would offer more time to give more fluid and blood product resuscitation, but also decrease the likelihood of demonstrating extravasation. Indeed, patients without extravasation had longer delays between the greatest decline in hematocrit and arteriogram. 

In this study, not all patients underwent endoscopy or CTA evaluation prior to angiography. Among those patients that did, neither endoscopic or CTA findings accurately predicted which patients would have active extravasation, despite prior work suggesting that CTA is accurate in detecting gastrointestinal and intraperitoneal bleeding [6,8]. The discordance in the findings presented here and previous reports could be due to bias in the selection of patients for endoscopy and CTA prior to arteriography. Alternatively, longer delays before angiography, allowing for endoscopic or CTA evaluation, could have reduced the likelihood of finding extravasation on angiography, as it is well established that GIB is intermittent and timing of procedures is critical to detect active bleeding [15]. 

When evaluating a patient for potential conventional angiography, one must also consider potential risks. Findings here are similar to prior studies with CIN being the most common documented adverse event (15%) [17,25]. CIN is a serious complication. In the intensive-care unit (ICU) setting, CIN is associated with increased hospital stay and higher 28-day mortality (36% for patients with CIN vs. 13% for patients who do not develop CIN) and 1-year mortality (56% vs. 24%) [26]. The risk of CIN varies from 1% to 27% depending on studies and risk factors, with chronic kidney disease being the most significant risk factor for subsequent development of CIN [17,25]. In patients with severe renal impairment, roughly 50% are reported to develop CIN [27], compared to the 67% reported here. Whereas fewer than 1% of patients with GFR > 60 and fewer than 3% of patients with GFR 30–60 are expected to have acute renal failure after contrast [28], we found 8% of patients with GFR > 60 and 29% of patients with GFR 30–60 developed CIN. Patients with acute bleeding are likely at higher risk of CIN given inherent concomitant hypovolemia and treatment with intravenous vasopressors. For instance, more than 15% of patients with sepsis in the surgical ICU, and more than 10% of cirrhotic patients with gastrointestinal hemorrhage develop acute renal failure [29,30], highlighting the elevated risk of renal failure in patients with hypotension and hemorrhage. The higher probability of CIN in the bleeding patients in this study compared with reported frequency of CIN in non-bleeding patients therefore likely reflects the added contribution of hypotension and hypovolemia.

The present study corroborates pre-existing renal dysfunction as an independent risk factor for the development of CIN after contrast administration [17,25]. GFR prior to conventional angiography was the only independent predictor of CIN, with a relatively large increase in risk with any decrease of GFR below 60. Type and amount of contrast used showed no correlation with subsequent development of CIN, which is consistent with more recent literature [31,32]. CHF was more common (40%) in patients who developed CIN than those who did not (18%). While this finding was not statistically significant, it corroborates prior reports that CHF is a risk factor for CIN [33], though other studies have not shown this association [34]. The findings reported here suggest that GFR is the best clinical variable to help triage potential high-risk patients for development of CIN. Patients with low GFR should prompt consideration of different evaluation procedures such as endoscopy or tagged RBC scans to avoid the possibility of CIN, particularly when ΔHct does not exceed 20%. 

The primary limitation is the retrospective nature of the study, introducing potential bias. There was also a relatively small sample size within the current study; however, this was partly counterbalanced by the accuracy and completeness of the electronic medical chart. With regards to evaluation of endoscopy and CTA as clinical predictors, not all patients underwent evaluation by these modalities, making it difficult to ascertain an accurate correlation between these modalities and a positive angiographic study. Furthermore, not all patients had follow-up creatinine assays 48–72 h after the procedure; the excluded patients could have been less ill, thus artificially increasing the rate of CIN detected here. Thus, the CIN rates reported here could be considered a conservative estimate. Future studies could address the long-term outcomes of patients who develop CIN after arteriography for active bleeding.

## 5. Conclusions

In conclusion, the ΔHct over 24 h was the best predictor of a positive angiographic study. When in doubt, a decline exceeding 20% should prompt a rapid angiographic study, as it is more than 50% likely that the patient will have active extravasation. When describing the risks of the procedure, CIN is the most likely adverse event. The CIN risks here, stratified by pre-existing GFR, may serve as a guide when educating and consenting patients. These findings can help interventional radiologists triage patients to maximize the likelihood of a positive angiographic study while minimizing the risk of subsequent development of CIN.

## Figures and Tables

**Table 1:** Univariate analysis comparing studies with and without active extravasation.

Variable	No Extravasation	Extravasation	*p* Value
*n*	55	20	
Age	60 (2.5)	57 (4.6)	0.59
Gender (% Male)	64%	60%	0.77
Urine output (mL) ^*^	1207 (169)	1067 (340)	0.32
pRBC (units)	1.6 (0.3)	0.5 (0.2)	0.02
Fluids (mL) ^*,**^	1870 (322)	1318 (424)	0.35
MAP ^+^	88 (2)	87 (2)	0.61
Pressor ^*^	45%	45%	0.97
HR ^+^	91 (2.4)	89 (3.9)	0.65
ΔHct ^*^	−1% (3%)	−17% (5%)	0.048

pRBC: packed red blood cells; MAP: mean arterial pressure; HR: heart rate; ΔHct: percent change in hematocrit over 24 h. ^*^ Values assessed in the 24-h period prior to angiography. ^**^ Fluids indicate all blood products and other fluids, except pRBC, given in resuscitation during the 24 h prior to examination. ^+^ Vital signs documented just prior to angiogram.

**Table 2:** Change in hematocrit over 24 h prior to angiography, for patients with suspected upper and lower gastrointestinal bleeding (GIB).

Change in Hematocrit (ΔHct)	Number of Studies	Active Extravasation
−40% to −60%	5	60%
−20% to −40%	8	50%
0% to −20%	17	12%
Any increase	16	19%

**Table 3:** Comparison of patients who developed contrast-induced nephropathy (CIN) to those who did not.

Variable	No CIN	CIN	*p* Value
*n*	56	10	
Gender (% Male)	64%	70%	0.73
Age (year)	62 (2.4)	65 (6.1)	0.67
pRBC (units)	1.4 (0.3)	1.3 (0.5)	0.89
Fluids (mL)	2180 (350)	1378 (608)	0.36
MAP	89 (1.8)	85 (3.0)	0.41
Pressor	55%	40%	0.37
HR	86 (1.9)	93 (6.4)	0.39
CHF	18%	40%	0.11
DM	13%	10%	0.47
Contrast (mL)	141 (9)	156 (19)	0.46
Contrast type ^*^	29%	40%	0.47
Extravasation	20%	30%	0.46
ΔHct	−5%	−20%	0.07
GFR	>60 (5)	57 (8)	0.02

*n*: number of subjects; MAP: mean arterial pressure; HR: heart rate; CHF: congestive heart failure; DM: diabetes mellitis; ΔHct: percent change in hematocrit over 24 h; GFR: glomerular filtration rate. ^*^ Percentage indicates the proportion of patients who received Visipaque (the remaining patients received Ultravist).

**Table 4:** Percentage of patients who developed CIN.

GFR	Number of Studies	CIN
<30	3	67%
30–59	14	29%
>60	49	8%

GFR: glomerular filtration rate, value assessed immediately prior to the angiogram; CIN: contrast-induced nephropathy.
